# Basal Ganglia Local Field Potentials as a Potential Biomarker for Sleep Disturbance in Parkinson's Disease

**DOI:** 10.3389/fneur.2021.765203

**Published:** 2021-10-28

**Authors:** Alexander J. Baumgartner, Clete A. Kushida, Michael O. Summers, Drew S. Kern, Aviva Abosch, John A. Thompson

**Affiliations:** ^1^Department of Neurology, University of Colorado School of Medicine, Aurora, CO, United States; ^2^Department of Psychiatry and Behavioral Sciences, Stanford University School of Medicine, Stanford, CA, United States; ^3^Department of Medicine, Division of Pulmonary, Critical Care, Sleep, and Allergy, University of Nebraska Medical Center, Omaha, NE, United States; ^4^Department of Neurosurgery, University of Colorado School of Medicine, Aurora, CO, United States; ^5^Department of Neurosurgery, University of Nebraska Medical Center, Omaha, NE, United States

**Keywords:** Parkinson's disease, sleep, local field potential (LFP), deep brain stimulation (DBS), biomarker

## Abstract

Sleep disturbances, specifically decreases in total sleep time and sleep efficiency as well as increased sleep onset latency and wakefulness after sleep onset, are highly prevalent in patients with Parkinson's disease (PD). Impairment of sleep significantly and adversely impacts several comorbidities in this patient population, including cognition, mood, and quality of life. Sleep disturbances and other non-motor symptoms of PD have come to the fore as the effectiveness of advanced therapies such as deep brain stimulation (DBS) optimally manage the motor symptoms. Although some studies have suggested that DBS provides benefit for sleep disturbances in PD, the mechanisms by which this might occur, as well as the optimal stimulation parameters for treating sleep dysfunction, remain unknown. In patients treated with DBS, electrophysiologic recording from the stimulating electrode, in the form of local field potentials (LFPs), has led to the identification of several findings associated with both motor and non-motor symptoms including sleep. For example, beta frequency (13–30 Hz) oscillations are associated with worsened bradykinesia while awake and decrease during non-rapid eye movement sleep. LFP investigation of sleep has largely focused on the subthalamic nucleus (STN), though corresponding oscillatory activity has been found in the globus pallidus internus (GPi) and thalamus as well. LFPs are increasingly being recognized as a potential biomarker for sleep states in PD, which may allow for closed-loop optimization of DBS parameters to treat sleep disturbances in this population. In this review, we discuss the relationship between LFP oscillations in STN and the sleep architecture of PD patients, current trends in utilizing DBS to treat sleep disturbance, and future directions for research. In particular, we highlight the capability of novel technologies to capture and record LFP data *in vivo*, while patients continue therapeutic stimulation for motor symptoms. These technological advances may soon allow for real-time adaptive stimulation to treat sleep disturbances.

## Introduction

The cardinal motor features of Parkinson's disease (PD) include bradykinesia, rest tremor, and rigidity. Though non-motor features have been recognized since the original description of the disease by James Parkinson in 1817, only recently has the prevalence and impact of non-motor symptoms become the focus of intense study (1, 2). Disturbances of sleep are among the most common non-motor manifestations of PD. In a 2009 survey of more than one thousand PD patients across 55 clinical centers, ~37% of patients reported experiencing insomnia, 21% reported excessive daytime sleepiness, 15% reported restless legs, and 30% reported rapid eye movement (REM) sleep behavior disorder (RBD) (3). In all, 64% of PD patients reported at least one symptom affecting sleep, which was second in frequency only to psychiatric symptoms (the most common being anxiety/nervousness, depression, and anhedonia, prevalence 67%). Sleep disorders commonly occur prior to the appearance of typical motor symptoms. The most well-known prodromal sleep disorder is RBD, which may develop years to decades prior to the onset of motor symptoms (4–6). The presence of RBD is one of the most specific predictors for developing a neurodegenerative disease, with a risk of over 90%. The risk for developing PD specifically, when RBD is present, may be as high as 65% (7–9).

Given that sleep contributes to the regulation of many physiological homeostatic processes, sleep disturbance has a significant impact on quality of life in PD (10–12), and places high strain on caregivers, even predicting earlier transfer to a nursing home (13–15). Thus, the ability to treat sleep disorders represents an opportunity to make substantial improvements in not only mood, cognition, and overall satisfaction, but also significantly alleviate caregiver burden and relieve financial strains associated with the need for nursing home care. Though numerous symptomatic therapies exist, the treatment of sleep disorders in PD is limited by a lack of adequately powered, randomized studies providing high quality evidence (16). The possibility of treating disorders of sleep with DBS is thus an appealing one. Although, DBS is primarily used to treat PD motor symptoms and reduce the need for dopaminergic medications, several studies have shown that DBS provides benefit for non-motor symptoms, including sleep disturbance (17, 18). However, the optimal DBS target and stimulation parameters to address sleep remain unknown. In recent years, recording of LFPs primarily from STN has identified unique spectral patterns in oscillatory activity between the awake, REM sleep, and non-REM (NREM) sleep states, thereby providing novel insights into sleep architecture and basal ganglia physiology in patients with PD. STN LFPs may therefore be suitable for use as a biomarker for sleep, allowing stimulation to be tailored to ameliorate sleep disturbance. This article will briefly discuss pathophysiology of sleep-wake disturbances in PD, review existing literature on subcortical electrophysiology in sleep, highlight the potential for novel DBS technologies to address sleep, and describe future directions for investigating the use of LFPs as a biomarker for treating sleep disturbance with DBS.

## Sleep Disturbances in PD

Sleep is classified into NREM and REM stages based on polysomnography (PSG), which is primarily comprised of electroencephalogram (EEG), electrooculogram (EOG), and electromyogram (EMG) recordings. NREM sleep is notable for slow, rolling eye movements, prominent parasympathetic tone, and rare dreaming (19, 20). NREM sleep is further divided into stages N1–N3. In N1, the lightest sleep stage, the normal waking posterior dominant alpha (8–12 Hz) rhythm is lost (21). N2 sleep is characterized by the emergence of both sleep spindles (brief oscillations of 12–14 Hz) and K-complexes (sharp high voltage biphasic waves lasting more than 0.5 s) (21). In N3 (slow-wave) sleep, delta (0.5–2 Hz) EEG waves make up at least 20% of any given sleep epoch (21). REM sleep is characterized by low-amplitude, mixed frequency, desynchronized EEG (similar to wakefulness with the eyes open), rapid eye movements, and suppressed chin EMG activity (21). Dreams typically occur during REM sleep (22).

Nearly all aspects of sleep are affected in PD, though disorders of sleep-wake transition/sleep architecture as well as parasomnias (i.e., atypical/unusual behaviors during sleep) affecting both REM and NREM sleep are more common (23–28). Understanding of these sleep disturbances has been largely driven by studies using formal PSG in case-control studies.

PSG studies of PD patients have demonstrated several alterations in sleep architecture, including increased sleep onset latency, reduced total sleep time, increased wakefulness after sleep onset (WASO), and decreased sleep efficiency (defined as the ratio of time asleep to time spent in bed) (29–33). Decreased sleep efficiency has been demonstrated in studies involving both treated and untreated PD patients (31, 34, 35). In investigating NREM sleep stages, studies have demonstrated a trend toward increased time spent in stage N1, an effect which seems to be most prominent in the early stages of PD (31, 35, 36). Alterations in the architecture of NREM sleep seem largely to be driven by changes during stages N2 and N3, both of which are reduced (37–39). It should be noted that these changes have not been universally reported, reflecting the likely heterogeneity of PD and resultant sleep disturbances, as well as methodological differences between studies (31, 35, 37, 40–42). However, a recent meta-analysis from 2020 did confirm a reduction in both N2 and N3 sleep in PD patients vs. control subjects (43). The reduction in N3 sleep, in particular, seems to be progressive in a manner that correlates with disease duration (30, 37).

Aberrant REM sleep is a consistent feature of PD. In the same recent meta-analysis mentioned above, the percentage of time spent in REM sleep and duration of contiguous REM epochs (i.e., REM density) were significantly reduced in PD patients compared to controls, while REM latency was increased (43). Decreased REM sleep has been found in *de novo*, untreated PD patients, and the duration of REM sleep seems to shorten with disease progression (30, 36). Furthermore, REM sleep is characteristically affected by RBD, a parasomnia characterized by abnormal behaviors, such as talking, laughing, shouting, gesturing, grabbing, flailing, punching, or kicking during REM sleep that are associated with dream content and enactment (44). Estimates of the prevalence of RBD in PD range from ~30 to 60% (45–47).

Electrophysiological alterations in sleep in PD occur across stages N2, N3, and REM. N2 sleep in PD seems primarily to be affected by a reduction in K-complexes and sleep spindle formation, a finding which has been replicated across several studies (29, 39, 48–50). A reduction in sleep spindle density has also been found in patients with idiopathic RBD (51). A single study, in contrast to these results, found no difference in the quantity of K-complexes or sleep spindles in PD patients compared to controls, though it should be noted that PD patients included in this study had lower Movement Disorders Society Unified Parkinson's Disease Rating Scale (MDS-UPDRS) scores and lower levodopa doses, raising the possibility that reduction in K-complexes and sleep spindles is not a feature of early, mild disease (32). Slow wave activity (SWA), an EEG oscillatory pattern of 0.5 to 4.0 Hz that is a normal hallmark of stage N3 sleep, is reduced in PD (31, 43, 45, 52, 53). This reduction may become more severe with advancing PD (52). REM sleep in PD patients is marked by increased power in the high theta/alpha frequency range (7.8–10.5 Hz) (40, 54, 55). This alteration in REM physiology has been mostly observed in early, untreated patients or in those not taking dopaminergic medications, and was not seen in a study of patients on dopaminergic therapy, raising the possibility that antiparkinsonian medications may modulate REM sleep (56). Further study of this hypothesis is needed.

Further discussion of the spectrum of sleep disorders in PD can be found in recent reviews by Chahine et al. (16) and Zhang et al. (43).

## Subcortical Electrophysiology and Relevant Pathways of Sleep—Animal Studies

Our understanding of the electrophysiological activity of subcortical structures has been greatly informed by studies of normal animals as well as animal models of PD. These studies have provided significant insight into mechanisms of, and potential therapies for, sleep disturbance in PD.

Studies in rodents using single-neuronal as well as multi-unit activity (MUA) recordings have demonstrated a rhythmic bursting pattern in STN neurons during slow-wave sleep, while globus pallidus neurons exhibited a slowing in firing rate during slow wave sleep compared to both wakefulness and REM (57, 58). During slow-wave sleep, medium spiny neurons of the striatum display brisk firing resulting from rhythmic membrane potential fluctuations, unlike the irregular and disorganized firing seen during wakefulness (59).

Recent animal studies exploring the generation and maintenance of sleep at the single neuron and circuit levels have revealed a multi-nodal brain-wide network contributing to the sleep-wake transition. These studies posit that control of sleep requires the integration and coordination of both autonomic and somatomotor control networks, with distinct circuits, including the basal ganglia, contributing to this global brain state (60). Several regions of the basal ganglia, specifically within the indirect pathway [inhibition of gamma-aminobutyric acid (GABA)-ergic neurons of the GPe, leading to disinhibition of glutaminergic neurons of the STN and thus activation of GABAergic substantia nigra pars reticulata (SNr) neurons projecting to the thalamus], have been implicated in different stages of sleep. Both the nucleus accumbens and dorsal striatum have been shown to promote NREM sleep *via* activation of adenosine A_2A_ receptor (A_2A_R)-expressing GABAergic neurons (61, 62). In particular, the A_2A_R neurons of the dorsal striatum were found to innervate the globus pallidus externus (GPe) in a topographical pattern synapsing onto parvalbumin (PV)-expressing neurons, and ablation of these PV neurons abolished the NREM-promoting effect of A_2A_R activation (62). In addition to these findings in GPe, glutamatergic neurons of STN projecting to the SNr, when activated *via* optogenetic manipulation, significantly prolong NREM states (63). Even within the zona incerta (ZI), which lies dorsal and posterior to the STN, LIM homeodomain factor (Lhx6)-expressing GABAergic neurons within the ventral ZI promote NREM sleep *via* selective activation, and decrease both NREM and REM sleep when selectively ablated (64). Finally, the substantia nigra, which is a basal ganglia output structure and a critical node within the indirect pathway, also contributes to sleep regulation: Optogenetic activation of glutamate decarboxylase 2 (Gad2) neurons within the medioventral region of SNr both terminates movement during wake periods and significantly enhances the initiation of sleep (65).

Mizrahi-Kliger et al. (66) studied the activity of single neurons in the basal ganglia in a pair of vervet monkeys. They found that the firing rate of basal ganglia neurons was significantly lower and more irregular (burst-like) during slow wave sleep than during REM and wakefulness. This was particularly true in GPi, GPe, and SNr. Basal ganglia neurons also exhibited slow oscillations in firing rate during slow wave sleep, similar to those observed in cortical neurons in both humans and non-human primates. LFP recordings in the basal ganglia demonstrate dramatically reduced slow oscillations compared with thalamocortical networks. Furthermore, unlike in the thalamus and cortex, basal ganglia LFPs were noted to be desynchronized between individual neurons. Proposed causes for this inter-neuronal desynchronization include the highly convergent nature of input to the basal ganglia, wherein a single GPi or GPe neuron may receive input from numerous striatal cells, each of which, in turn, is receiving input from numerous cortical cells (67). Thus, the basal ganglia are uniquely placed in brain-wide sleep physiology, by virtue of receiving slow oscillatory activity from vast cortical areas and, *via* dyssynchronous firing, capable of activating multiple disparate cortical areas at any one time.

In the 1-methyl-4-phenyl-1,2,3,6-tetrahydropyridine (MPTP) primate model of parkinsonism, increased power in an LFP frequency band that encompassed alpha and low beta (10–17 Hz) activity during NREM was seen in GPe, GPi, and STN (68). This increase in alpha and low beta activity was associated with a decrease in the power of slow oscillatory firing of the basal ganglia. Epochs with higher average beta power were associated with a decreased propensity for sleep and an increased frequency of awakenings. Furthermore, the authors demonstrated a temporal association between beta activity and sleep-wake cycles, in that falling asleep was associated with a gradual decrease in LFP beta activity across the basal ganglia, and beta oscillations became more prominent in the approach to awakenings. Thus, given the results of these studies, a potential mechanism for sleep disturbance in PD emerges, whereby synchronized beta oscillations from the basal ganglia are relayed to the cortex, disrupting cortical slow oscillations that are characteristic of NREM sleep (66, 68). Of course, non-human animal studies should not provide the sole basis for informing conclusions about human physiology. Although MPTP does produce an excellent model of parkinsonism, the disease course is more fulminant in the model—both in rapidity of onset and in severity—than in idiopathic PD. Additionally, sleep in PD is almost certainly affected by degeneration of several different brain nuclei and neurochemical pathways not targeted by MPTP, which is highly selective for dopaminergic neurons (69). Collectively, these shortcomings of the MPTP model may preclude a faithful recapitulation of the progressive nature of sleep dysfunction in patients with PD.

## Subcortical Electrophysiology and Relevant Pathways of Sleep—Human Studies

In PD patients, early studies using MUA demonstrated slow oscillations in the globus pallidus and caudate during slow wave sleep, similar to those identified in primates (70). These oscillations were similarly attenuated during wakefulness and REM sleep. In a study of PD patients with STN DBS, single-unit recordings demonstrated a decrease in firing rate during sleep, and that neuronal firing developed a grouped or bursting pattern (71).

More recently, electrophysiological studies have focused on LFP recordings rather than on single or multi-unit data (Table 1). This was largely driven by several important characterizations of LFPs, primarily in PD patients: First, LFP activity is strongly correlated with several PD disease states, in particular the OFF state, in which power in the beta band (typically 13–30 Hz) is prominent in both STN and GPi (76–80). Specifically, increased beta frequency band power is associated with worsened bradykinesia and rigidity, but not with tremor (79–81). After administration of dopaminergic medications, beta activity attenuates and other frequencies, for instance 4–10 and 60–90 Hz, become more prominent (76, 78, 79, 82, 83). Not all studies, however, have demonstrated the 60–90 Hz peak after administration of medication (84). There is also an association between specific LFP bands and specific PD symptoms. For instance, decreased power in the high beta range (20–30 Hz) and increased power at <10 Hz have been reported in PD patients with dyskinesia (85). Furthermore, LFPs are an appealing target of study as they reflect synchronous changes in large populations of neurons, are locally generated (rather than conducted from the cortex), and are suppressed by both behaviorally relevant stimuli and voluntary movement (81).

**Table 1:** Summary of pertinent studies of LFPs in sleep in PD patients.

**Study**	**Sample size, *n***	**Age, mean (range)**	**PD duration, mean (range)**	MDS-UPDRS part III^*^, mean (range)	**Location**	**Timing (after DBS implantation)**	**Medications**	**Recording period analyzed**	**Major findings**
Urrestarazu et al. (72)	10	62.2 (56–72)	12.3 (8–25)	34.4 (26–46)	Bilateral STN	2–4 days	Off (timing not specified)	3 min sleep + 3 min wakefulness (5 afternoon naps, 5 nocturnal sleep)	NREM: beta power reduced compared to OFF-period wakefulness
									REM: high beta (20–30 Hz) power slightly higher power than wakefulness; low beta (13–20 Hz) lower than wakefulness
									RSWA: beta power higher than REM with atonia
Thompson et al. (73)	10	58.4 (39–70)	UA	40.4 (15–62)	Unilateral STN	3 weeks	Off several hours	24-h recording; average 7.5 h nocturnal sleep	NREM: increased power in delta, theta, alpha range, decreased beta, and gamma power compared to wakefulness
									REM: beta power increased but lower than wakefulness
									Beta power increased over the course of the night
									Subject-specific models able to classify sleep stage based on LFP signature
Chen et al. (74)	12	54.8 (40–67)	10.2 (7–20)	UA	Bilateral STN	1 month	Off at least 8 h	4–6 h nocturnal sleep; 6 min wakefulness prior to sleep	NREM: increased power in delta, theta, alpha range, decreased beta, gamma power compared to wakefulness
									REM: beta, gamma power increased, similar to wakefulness
									Subject-specific models able to classify sleep stage based on LFP signature

* *Before DBS, off medication*.

*PD, Parkinson's disease; MDS-UPDRS, Movement Disorders Society-Unified Parkinson's Disease Rating Scale; STN, subthalamic nucleus; DBS, deep brain stimulation; NREM, non-rapid eye movement sleep; REM, rapid eye movement sleep; RSWA, REM sleep without atonia; UA, unavailable; ANN, artificial neural network*.

In the first study using LFPs to assess sleep, Urrestarazu et al. (72) recorded LFPs from STN in 10 PD patients undergoing DBS implantation. Recording was performed between 2 and 4 days after macroelectrode implantation. Notably, in five subjects, sleep recordings were acquired during nap periods in the early afternoon, and in the remaining five subjects, were acquired during nighttime sleep. For each subject, analysis was conducted on a total of 6 min accumulated from 18 artifact-free segments, each of 10-s duration and drawn from each sleep and awake period (i.e., 3 min acquired during periods of wakefulness and 3 min acquired during sleep). During NREM sleep, power in the beta frequency band was significantly reduced compared to OFF-state wakefulness. In five patients from whom REM sleep was recorded, beta activity, particularly at 20–30 Hz, again became more prominent and in fact occurred at a slightly higher power than in wakefulness (Table 2). Low beta (13–20 Hz) power was lower in REM compared to wakefulness. Four of the five patients additionally showed a peak at 10–15 Hz, which was not seen during other sleep stages nor during wakefulness. In three patients who exhibited REM sleep without atonia (RSWA), power in the beta band was higher during RSWA episodes compared with episodes of REM with atonia. No video assessment of movement was made, so it is unknown if these episodes of RSWA were associated with dream enactment behaviors suggestive of RBD.

**Table 2:** Characteristic LFP signatures of sleep and wakefulness in PD.

**State**	**LFP characteristics**	**Notes**
Wakefulness—OFF state	↑ Beta frequency power	Beta frequency power more closely linked with bradykinesia, rigidity than tremor
Wakefulness—ON state	↓ Beta frequency power	Emergence of gamma frequency not consistent across studies
	↑ Theta, gamma frequency power	Dyskinesia may be associated with increased theta and decreased high beta (20–30 Hz) power
NREM sleep	↑ Delta, theta, and alpha frequency power	Significant between-patient variability in relative power across frequencies
	↓ Beta frequency power	Most studies do not differentiate between stage N1, N2, and N3 sleep
REM sleep	↑ Beta frequency power	Beta frequency power may be lower, similar to, or greater than in wakefulness
	↓ Delta, theta, and alpha frequency power	Beta power may be higher in periods of REM without atonia than in REM with atonia

*NREM, non-rapid eye movement sleep; REM, rapid eye movement sleep*.

*Canonical frequency bands are as follows: delta (0–3 Hz), theta (3–7 Hz), alpha (7–13 Hz), beta (13–30 Hz), and gamma (30–90 Hz). ↑, Increased; ↓, decreased*.

**Figure 1:**
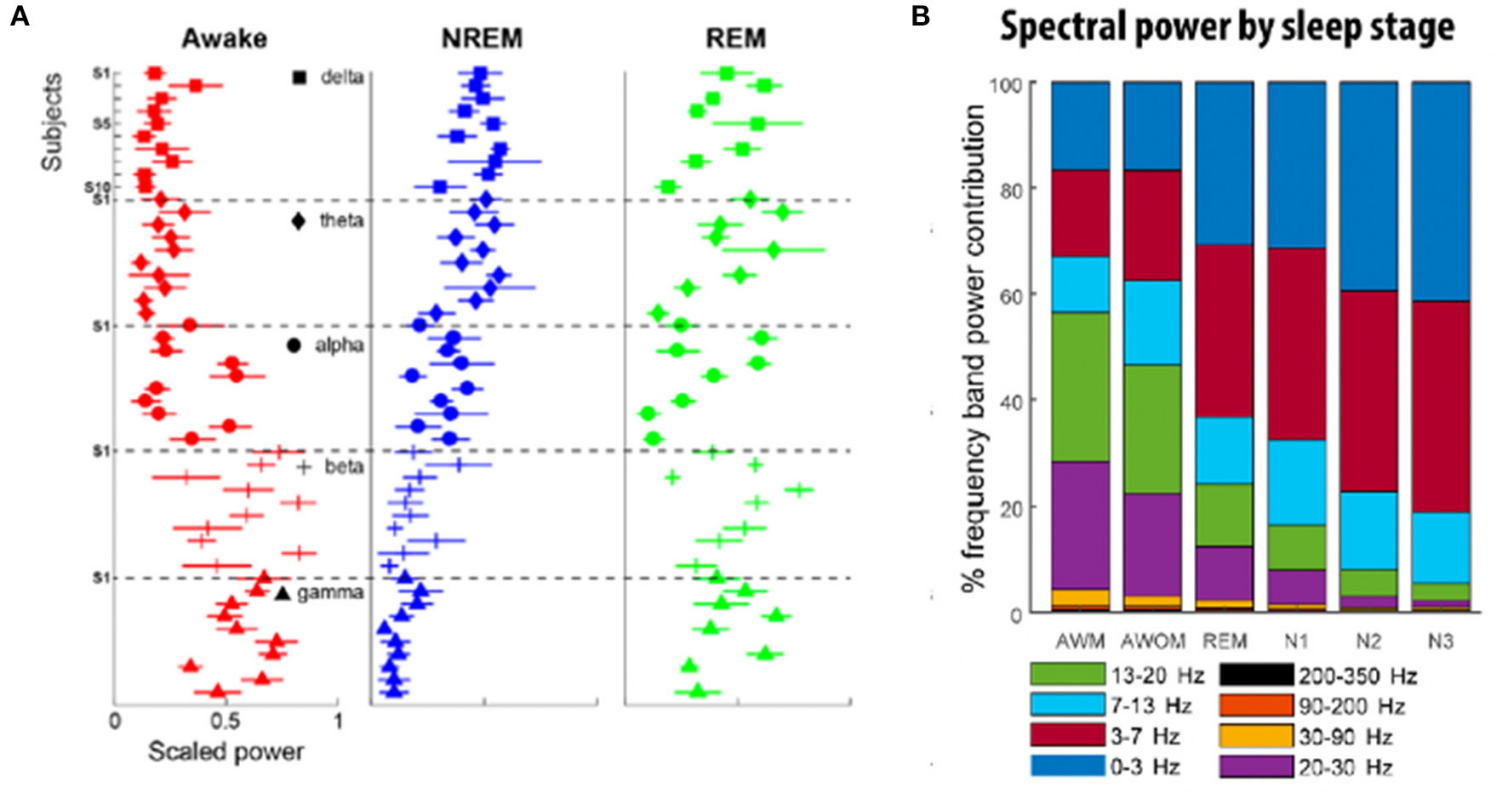
**(A)** Relative frequency contribution of each spectral band to different sleep stages. There exist shared sleep-stage dependent spectral patterns across subjects, although with some notable across-subject variability. Each individual plot highlights the distribution of the power of a given frequency band to different stages of sleep for 10 different subjects. In the awake state (red), power is highest in the beta and gamma frequencies, while NREM sleep (blue) is dominated by lower frequencies (delta, theta, and alpha). REM sleep (green) exhibits the greatest variability in representation across the frequency spectra [adapted from (73)]. **(B)** Distribution of frequency band power contribution to sleep stage for a cohort of nine subjects. AWM, awake with movement; AWOM, awake without movement; REM, rapid eye movement; N1–3, non-rapid eye movement stages 1–3 [adapted from (75)].

**Figure 2:**
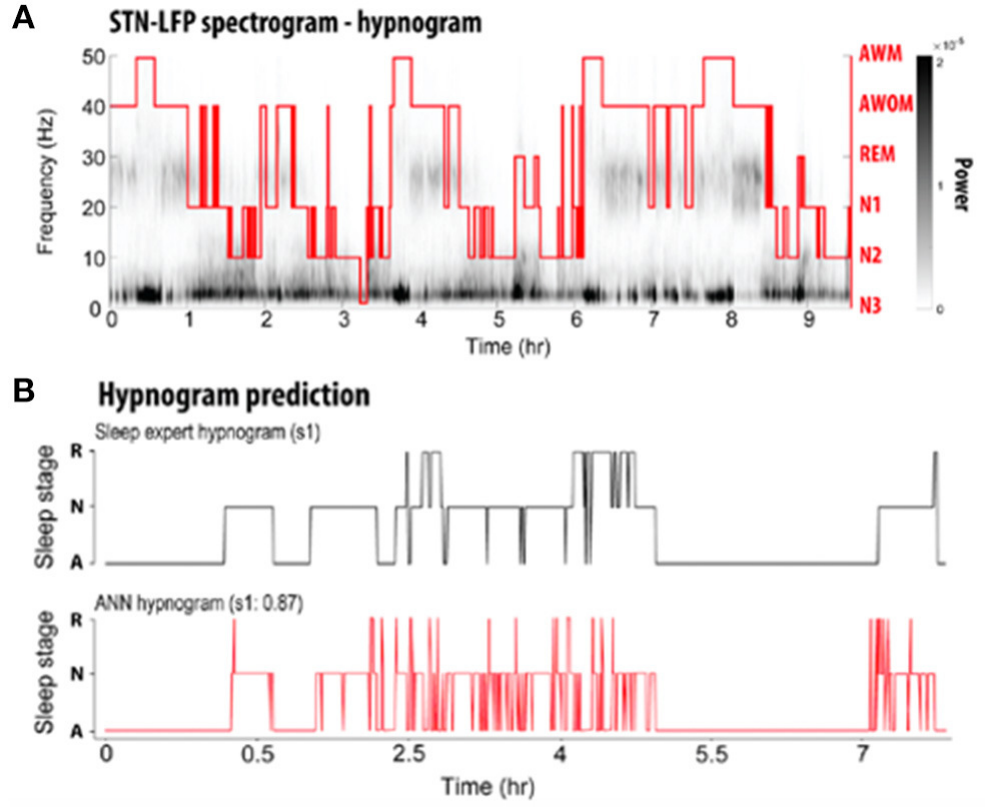
**(A)** Representative spectrogram of a LFP recording acquired over the course of one full night's sleep from a DBS electrode implanted into the STN. A PSG-informed hypnogram assessed by a sleep expert is aligned with the LFP recordings (red line). AWM, awake with movement; AWOM, awake without movement; REM, rapid eye movement; N1–3, non-rapid eye movement stages 1–3. **(B)** Comparison of hypnogram assessed by a sleep expert (top; black) and ANN-predicted hypnogram (bottom; red) from a single patient. R, rapid eye movement; N, non-rapid eye movement; A, awake [adapted from (75)].

Subsequent studies have demonstrated the feasibility of using LFPs to determine sleep stage. Thompson et al. (73) recorded LFPs from the STN of 10 patients 3 weeks after implantation of a DBS macroelectrode for a single, full night of sleep (~9 h per subject), and compared these to formal PSG obtained concurrently. Polysomnography was scored according to the 2007 American Academy of Sleep Medicine (AASM) guidelines (86). These investigators found significant band-power differences in all NREM states compared with REM and wakefulness, in a manner that corroborates typical EEG findings. Delta (0–3 Hz), theta (3–7 Hz), and alpha (7–13 Hz) all increased in NREM sleep compared to REM, while beta and gamma (30–90 Hz) power decreased (Figure 1). In contrast to the findings of Urrestarazu et al., beta power during REM sleep was lower than in wakefulness. Importantly, there was significant between-subject variability in the relative power of each frequency band during each sleep stage, suggesting that an individualized analysis for each subject is likely necessary to accurately monitor and treat sleep dysfunction. To this end, the authors used a support vector machine (SVM) model to predict sleep stage based on LFP power spectra. SVM models accurately predicted sleep stage for the subject on which they were trained, but performed poorly for other subjects. This work was then improved by development of a feedforward artificial neural network (ANN) to predict sleep stage based on 30-s epochs of LFP data (Figure 2) (75). In a leave-one-group-out analysis, the ANN was able to predict sleep stage (awake, NREM, or REM, without breaking down NREM into component stages N1–3) with an overall accuracy of 91%, though accuracy was lower (77%) for REM sleep—likely a consequence of diminished REM in these PD study subjects. This represents a significant improvement in performance over the SVM, in that the ANN was able to accurately stage sleep in subjects it had not previously encountered.

Chen et al. (74) studied 12 PD patients undergoing STN DBS. LFPs were recorded at 1 month after electrode implantation and compared to PSG, staged according to AASM guidelines. They again demonstrated that delta, theta, and alpha band power significantly increased from wakefulness to N2 and decreased in REM sleep. Beta and gamma power decreased from wakefulness to N2. In REM sleep, beta band power was similar to wakefulness. As with Thompson et al., there was significant between-subject variability in relative power across frequency bands during each sleep stage. Machine learning algorithms were then applied to classify sleep stage based on LFP power spectra. Subject-specific models performed significantly better than a study-wide model. Classification accuracy was over 90% for distinguishing wakefulness from N1, wakefulness from N2/N3, wakefulness from REM, N1 from N2/N3, N2/N3 from REM, and wakefulness from sleep overall. Performance was lower (73%) for distinguishing N1 from REM sleep. A predictive model achieved similar accuracies.

Several important limitations to these studies should be noted: sample sizes were small, relatively heterogenous populations were included, and control groups were lacking for comparison. Given that the studies cited above involved externalized DBS leads for recording, data collection was restricted to a single night, thus limiting our knowledge of the significance of between-night differences in individuals. Recordings were also acquired in a hospital or sleep laboratory setting, creating an unfamiliar environment that likely affected naturalistic sleep behavior. Experiments were carried out between 2 days and 1 month following DBS implantation, making it difficult to know with certainty whether microlesional effects remaining from surgery or other peri-procedural factors influenced the results. However, it has been shown that the correlation between beta oscillations and parkinsonian syndromes during wakefulness remains present months and even years post-operatively, making it at least plausible that the same holds true for sleep (87, 88).

Though these studies provide support for a relationship between basal ganglia LFPs and sleep disturbance, important questions remain unanswered. First, it is unclear how to reconcile the increase in beta activity during REM sleep with the observation that motor control might actually be improved during REM (89). This increase in beta activity during REM would be unexpected given the otherwise akinetic nature of beta activity and may suggest a yet-undiscovered relationship between basal ganglia oscillations and movement. An alternate explanation for this apparent paradox might stem from the hypothesis that movement during REM sleep (i.e., dream enactment behavior) may bypass extrapyramidal pathways entirely (89). Additionally, the variability in beta power observed both between studies and within individuals over the course of a single night requires further exploration. As mentioned above, while some studies have found beta power in REM sleep to be similar to or slightly lower than during wakefulness, others have actually reported increased beta power during REM (72, 73, 75). In individuals, there does seem to be a reliable increase in beta activity as the night goes on, across all sleep stages (73). Thompson et al. speculate that these findings might be explained by the wearing-off of dopaminergic medications from last dose before bedtime until first dose of the morning. There might also be a relationship with REM atonia, as the study by Urrestarazu et al. (72) found a difference in beta power between episodes of RSWA and times of normal REM atonia. This finding should be interpreted with caution, as not all studies have corroborated it, and other mechanistic explanations, such as interaction with subcortical pontogeniculo-occipital (PGO) waves, have been posited (20, 90). Finally, the significance of LFPs in other subcortical structures, particularly in relation to sleep, is largely unknown. LFPs recorded from the centromedian-parafascicular nucleus of the thalamus in PD patients show a prominent band of gamma activity in the ON-state that disappears in the OFF-state, but there is as yet no known correlation with arousal state (91). The GPi also displays beta frequency oscillations that modulate with volitional movement, though again studies of GPi LFPs in sleep are lacking (92, 93).

## Impact of Conventional DBS on Sleep

**Figure 3:**
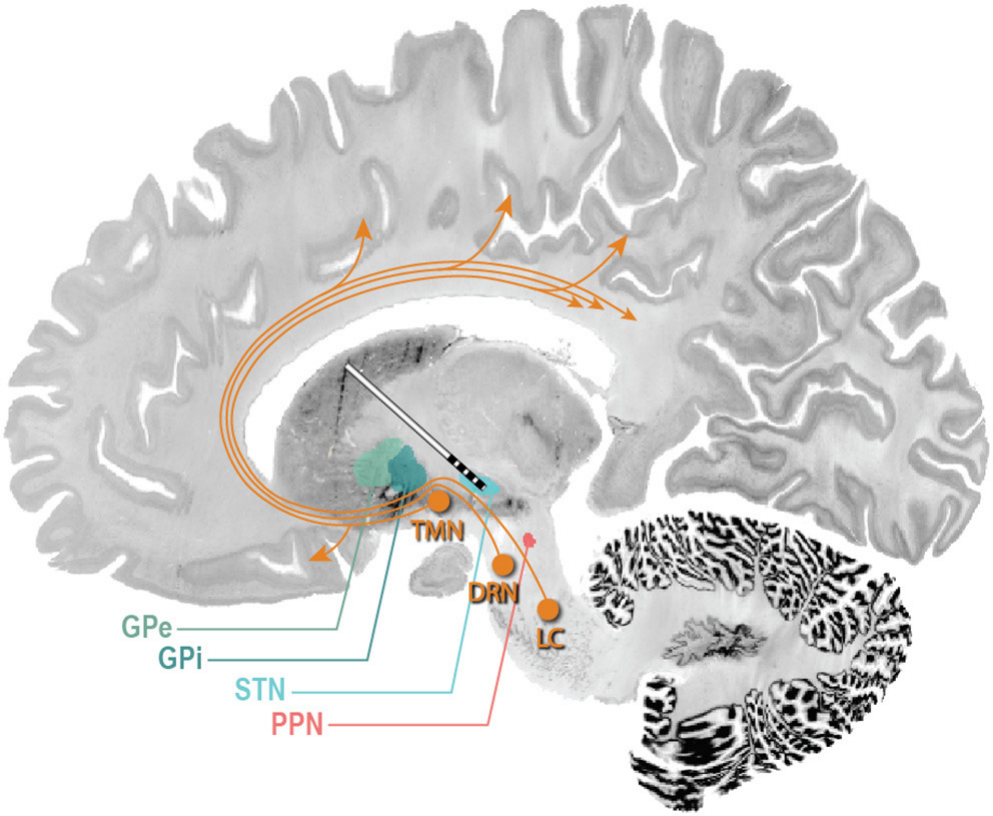
Potential DBS targets for treatment of sleep dysfunction in PD. STN DBS may increase total sleep time and sleep efficiency, reduce wakefulness after sleep onset, and in some studies, increase REM duration. GPi DBS may improve sleep quality and daytime sleepiness. A single study in five PD patients with GPi DBS demonstrated a non-statistically significant increase sleep quality and efficiency, with decreased WASO, sleep onset latency, and REM latency. GPe DBS may improve insomnia and improve sleep efficiency. PPN DBS may improve sleep efficiency, REM duration, and daytime sleepiness, and decrease WASO. The ascending arousal system (orange arrows) sends projections from the brainstem and posterior hypothalamus throughout the forebrain (116). Neurons of the laterodorsal tegmental nuclei and PPN send cholinergic fibers to many forebrain targets, including the thalamus, which then regulate cortical activity. Aminergic nuclei diffusely project throughout much of the forebrain, regulating the activity of cortical and hypothalamic targets directly. These include neurons of the tuberomammillary nucleus containing histamine, neurons of the dorsal raphe nuclei containing 5-HT, and neurons of the locus coeruleus containing noradrenaline. TMN, tuberomammillary nucleus; DRN, dorsal raphe nucleus; LC, locus coeruleus (117, 118).

The ability to record and stage sleep *via* STN LFPs raises the exciting possibility of using DBS to optimize treatment of not only the motor symptoms of PD during sleep (when patients are between medication doses), but also the disordered sleep itself. This possibility is bolstered by the observation that DBS, although not directly targeted nor programmed to improve sleep dysfunction, may confer some benefit on sleep in PD patients (94, 95). The evidence is most robust for STN DBS, where studies using subjective measures (validated sleep questionnaires) as well as objective measures (PSG or actigraphy) have demonstrated improvements in multiple sleep architecture outcomes including increased total sleep time and sleep efficiency, reduced wakefulness after sleep onset, and in some studies, an increase in REM sleep (18, 96–101). These improvements in subjective and objective measures seem to be an effect of stimulation, as studies examining sleep both on and off stimulation have demonstrated significant improvement in sleep when stimulation is on vs. off (17, 102). In addition to improvements in sleep architecture, STN DBS likely improves sleep *via* amelioration of nocturnal motor symptoms and may also improve symptoms of RLS, even when levodopa equivalent doses are reduced post-operatively (103–105). Current evidence does not suggest that STN DBS has any impact on RBD, though studies are limited (106). Evidence for the benefit of GPi DBS on sleep disturbance is sparse, though a few studies using subjective outcomes have suggested an improvement in sleep quality and daytime sleepiness with GPi DBS (107–109). A single study using objective sleep data in five PD patients with GPi DBS demonstrated a non-statistically significant increase sleep quality and efficiency, with decreased WASO, sleep onset latency, and REM latency (110). In a recent, double-blind, prospective, single-case report investigating the efficacy of GPe DBS for the treatment of insomnia, a PD patient with prolonged (7 years), severe insomnia, refractory to three hypnotic treatments, was implanted with one DBS lead in GPi and a second DBS lead in GPe. The patient exhibited improved sleep quality and decreased insomnia when GPe was selectively stimulated vs. co-stimulation of GPi and GPe (111). The study authors prospectively targeted GPe to ameliorate sleep disturbances based on prior animal studies that indicated activation of GPe resulted in increased REM and NREM duration (112, 113). Other targets have been suggested as possibly beneficial for sleep, particularly the pedunculopontine nucleus (PPN), given its role in sleep-wake modulation (114). In a series of five patients with both STN and PPN DBS, PPN stimulation improved sleep onset and maintenance insomnia compared to STN DBS. At 3 and 12 months, daytime sleepiness was improved by PPN DBS, but not by STN DBS (115). Further studies with larger numbers of patients are needed to accurately determine the efficacy of PPN DBS for sleep. Potential DBS targets for the modulation of sleep are highlighted in Figure 3. A recent review of these targets is provided by Sharma et al. (95).

## Utilizing LFPs for Adaptive DBS to Treat Sleep Disturbance

**Figure 4:**
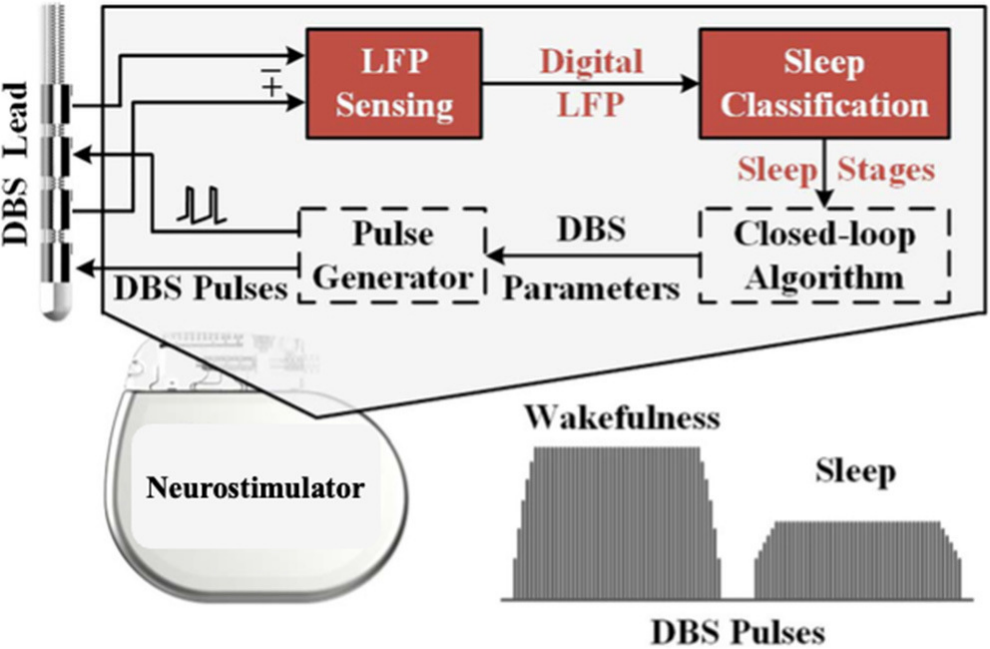
Schematic illustration of an adaptive closed-loop DBS system used to treat sleep dysfunction. LFPs are detected by the DBS lead. With integrated classifiers, sleep stages are predicted, and closed-loop algorithms can adjust the DBS pulses. For example, stimulation amplitude may be decreased during certain sleep stages where beta frequency power is lower [modified from (74)].

Adaptive DBS (aDBS) refers to a system wherein stimulation parameters are modulated in response to an inferred state of pathophysiological activity (119). A current challenge for adaptive DBS is inferring the pathological state, which currently relies on either peripheral measures, for instance tremor amplitude in a limb, or direct recording of brain activity. aDBS is an active area of research as these proxy pathophysiological measures are either imprecise (peripheral measures) or lack evidence (i.e., direct recordings from the brain). aDBS has primarily been pursued in an effort to widen the therapeutic window for treating motor symptoms and also to reduce power consumption of the implanted pulse generator (IPG). However, given the ability to accurately record sleep-wake cycles with basal ganglia LFPs (overcoming the evidence challenge) and the plausibility of treating sleep with DBS, sleep disturbance may be an ideal target for aDBS. A schematic illustration is shown in Figure 4.

A few important factors must be considered in the effort to use LFPs as a biomarker for treating sleep with aDBS. First, for any biomarker to be useful in the long term, it must be durable, that is, present for the duration of the disease or as long as the aDBS system is to be used. Little is known about any change or diminution of LFPs over time, though a small number of studies have demonstrated that LFPs can reliably be detected through implanted DBS leads as far as 7 years after DBS implantation (88, 120). Further studies will be needed to verify these results and to ensure that LFPs remain present during both wakefulness and sleep.

Second, the biomarker should correlate with symptom severity. Though beta activity is reliably present during certain sleep stages, as well as in times of increased bradykinesia and rigidity during wakefulness, it remains unknown whether beta activity is causally related. In the case of motor symptoms there is evidence that the severity of bradykinesia and rigidity, though not the presence or severity of tremor, correlate with LFP power in the beta band (79, 121). Thus, although a causal link is not fully established, it can at least be reasoned that reducing beta LFP power *via* the delivery of stimulation will lead to symptom improvement. The same correlation between symptom severity and LFP power has not been established for sleep disturbance, though in a nonhuman primate MPTP model of PD, a correlation between insomnia severity and beta power has been demonstrated (68). If also present in humans, this correlation might help explain the significant variability in beta frequency power between subjects observed in studies thus far (73, 74). Establishing a correlation between LFP power and insomnia severity in humans would be greatly facilitated by recording several continuous nights of sleep in individuals, so that between-night differences in LFP signature and symptoms could be examined. The development and recent FDA approval of the Medtronic Percept™ PC device now allows for the capturing and recording of LFPs simultaneously with the delivery of therapeutic stimulation for up to 60 days of stored data. This eliminates the need to obtain LFPs from an externalized DBS lead and will allow data to be collected in the home environment.

When considering aDBS for the treatment of sleep dysfunction, an optimal measure for efficacy must be determined. Should PSG studies be undertaken to examine changes in sleep architecture? This would provide the most robust evidence of a benefit from aDBS, though it would also be the most time-consuming and expensive. Furthermore, a single night of recording may be insufficient, especially as the mechanism by which DBS exerts its therapeutic effect remains unknown, but may involve changes in synaptic plasticity and long-term anatomical reorganization (119, 122). Validated sleep questionnaires would be much easier to administer, although these do not provide direct information on changes in sleep architecture. Care must be taken to ensure that outcomes of interest, such as insomnia and daytime sleepiness, are differentiated from other sleep-related symptoms which may not as clearly respond to DBS, such as restless legs syndrome (RLS) and obstructive or central sleep apnea (95). Actigraphy or other wearable devices may represent a useful intermediary between PSG and questionnaires. These technologies provide an objective measure of movement which may correlate to actual sleep. They are less expensive than PSG and can be used home setting. They have also been validated in both healthy controls and PD patients, to record accurately several sleep parameters including total sleep time, sleep efficiency, WASO, and nocturnal motor activity (123–126).

Finally, other contributors to sleep disturbance may pose significant challenges in using DBS, either through adaptive or conventional stimulation, to treat sleep. While DBS may be able to suppress or alter pathological oscillations in the basal ganglia, degeneration in other brain areas not amenable to DBS may play a role in sleep disturbance. Synuclein pathology is more prevalent in brainstem and hypothalamic sleep/wake centers, including the locus coeruleus, raphe nuclei, paramammillary nuclei, and posterior hypothalamus, in PD patients with sleep disorders compared to those without, suggesting that these areas likely play a critical role in sleep dysfunction (127). A myriad of other comorbidities may also contribute to sleep fragmentation. These include depression, pain, nocturia, and RLS, all of which are found in higher frequency in PD patients compared to controls (16, 128). The influence of dopaminergic medications adds another layer of complexity to sleep disturbances in PD. As mentioned above, wearing off of medications during the night may contribute to higher beta power over the duration of nocturnal sleep. Other medications may contribute to daytime sleepiness, as with dopamine agonists, or may worsen insomnia, as in the case of the monoamine oxidase inhibitor selegiline (16, 129). The potential confounding effect of these treatments will need to be carefully considered in designing future studies, particularly as greater emphasis is placed on studying sleep in the home setting over several nights.

## Conclusion

DBS is a highly effective therapy for the motor symptoms of PD. In recent years, the effect of DBS on non-motor PD symptoms has been investigated with increasing interest. Disorders of sleep are among the most prevalent non-motor symptoms of PD and come at a great cost to quality of life. The available evidence suggests that DBS does have a beneficial effect on sleep, specifically increased total sleep time and sleep efficiency, with reduced wakefulness after sleep onset. Subjective sleep measures are also improved by DBS. However, the optimal stimulation target and parameters to treat sleep, as well as the mechanisms by which DBS exerts its influence on sleep, remain largely unknown. Utilizing DBS to treat sleep disturbance will likely only be possible if a reliable biomarker for sleep exists. The most likely candidate biomarkers are LFPs. LFPs can reliably be recorded from the STN of PD patients, and multiple studies have proven the feasibility of using LFPs to determine sleep stage. Thus, LFPs would likely provide an excellent signal for an adaptive DBS system which targets sleep disturbance by varying stimulation in response to changes in sleep-wake state throughout the night. Additional research is needed to better define between-night differences in LFP signatures in individuals, establish a correlation between LFP power and symptom severity, and to develop and test aDBS systems aimed at treating sleep. If successful, these systems would likely have a profound impact on not only sleep, but also mood, cognition, and quality of life.

## Author Contributions

AB, DK, AA, and JT contributed to conception and design of the study. AB wrote the first draft of the manuscript. CK and MS provided key technical expertise and revisions. All authors contributed to manuscript revision, read, and approved the submitted version.

## Funding

This work was funded by NIH/NINDS grant UH3NS113769A1 and a University of Colorado intradepartmental grant.

## Conflict of Interest

The authors declare that the research was conducted in the absence of any commercial or financial relationships that could be construed as a potential conflict of interest.

## Publisher's Note

All claims expressed in this article are solely those of the authors and do not necessarily represent those of their affiliated organizations, or those of the publisher, the editors and the reviewers. Any product that may be evaluated in this article, or claim that may be made by its manufacturer, is not guaranteed or endorsed by the publisher.
